# Treatment with Cefotaxime Affects Expression of Conjugation Associated Proteins and Conjugation Transfer Frequency of an IncI1 Plasmid in *Escherichia coli*

**DOI:** 10.3389/fmicb.2017.02365

**Published:** 2017-11-29

**Authors:** Thea S. B. Møller, Gang Liu, Anders Boysen, Line E. Thomsen, Freja L. Lüthje, Sisse Mortensen, Jakob Møller-Jensen, John E. Olsen

**Affiliations:** ^1^Department of Veterinary and Animal Sciences, Faculty of Health and Medical Sciences, University of Copenhagen, Copenhagen, Denmark; ^2^Department of Biochemistry and Molecular Biology, University of Southern Denmark, Odense, Denmark

**Keywords:** antibiotic resistance, plasmid transfer, conjugation, Tra-proteins, *Escherichia coli*

## Abstract

Horizontal gene transfer (HGT) is the major mechanism responsible for spread of antibiotic resistance. Antibiotic treatment has been suggested to promote HGT, either by directly affecting the conjugation process itself or by selecting for conjugations subsequent to DNA transfer. However, recent research suggests that the effect of antibiotic treatment on plasmid conjugation frequencies, and hence the spread of resistance plasmids, may have been overestimated. We addressed the question by quantifying transfer proteins and conjugation frequencies of a *bla*_CTX−M−1_ encoding IncI1 resistance plasmid in *Escherichia coli* MG1655 in the presence and absence of therapeutically relevant concentrations of cefotaxime (CTX). Analysis of the proteome by iTRAQ labeling and liquid chromatography tandem mass spectrometry revealed that Tra proteins were significantly up-regulated in the presence of CTX. The up-regulation of the transfer machinery was confirmed at the transcriptional level for five selected genes. The CTX treatment did not cause induction of the SOS-response as revealed by absence of significantly regulated SOS associated proteins in the proteome and no significant up-regulation of *recA* and *sfiA* genes. The frequency of plasmid conjugation, measured in an antibiotic free environment, increased significantly when the donor was pre-grown in broth containing CTX compared to growth without this drug, regardless of whether *bla*_CTX-*M-*1_ was located on the plasmid or *in trans* on the chromosome. The results shows that antibiotic treatment can affect expression of a plasmid conjugation machinery and subsequent DNA transfer.

## Introduction

Plasmid conjugation contributes significantly to horizontal spread of antibiotic resistance, because antibiotic resistance genes are frequently located on plasmids (Zatyka and Thomas, 1998; Sorensen et al., 2005; Thomas and Nielsen, 2005; Bennett, 2008; Norman et al., 2009). However, whether antibiotic treatment increases the transfer rate *per se* is debatable. This question is not easily answered. A recent publication has reviewed a number of published conjugation experiments and pointed to the fact that the typical experimental setup cannot distinguish between increased transfer efficacy and selective growth advantage for the transconjugants in the presence of the antibiotic (post transfer). This report concluded that antimicrobials only serve to select for transconjugants, and that sub-lethal concentrations of antibiotics from the most widely used antimicrobial classes do not significantly increase the conjugation efficiency (Lopatkin et al., 2016a).

Beaber et al. (2004) reported that induction of the SOS-response by either mitomycin C or ciprofloxacin treatment enhanced the transfer of SXT, an integrative and conjugative element, which encodes antimicrobial resistance in *Vibrio cholerae* (Waldor et al., 1996), and in *Staphylococcus aureus*, the transfer of a resistance-plasmid carrying tetracycline resistance was reported to be greatly enhanced by the presence of sub-inhibitory concentrations of β-lactam antibiotics, also due to induction of the SOS-response (Barr et al., 1986). Similarly, treatment with sub-inhibitory concentrations of several antibiotics has been shown to induce the expression of conjugative transfer genes from an R-plasmid in *Aeromonas hydrophila* (Cantas et al., 2012), and low concentrations of tetracycline has been reported to increase transfer of conjugative transposons in strains of *Bacteroides* by a factor 10–100 (Whittle et al., 2002).

Plasmid conjugation in Gram-negative bacteria primarily occurs via type IV secretion (T4S), where single stranded plasmid DNA is transported between two cells forming a mating pair. The transfer is mediated by a multi-protein complex extending across the cell envelope of both the donor and recipient cells (Goessweiner-Mohr et al., 2013; Christie et al., 2014). Generally it is composed of ATPases, translocon proteins of the inner membrane, core proteins spanning the cell envelope and pilus proteins (Koraimann and Wagner, 2014). The IncF plasmid-group is a model for conjugative plasmids. In this group, more than 25 transfer genes (*tra* genes) are organized in a single continuous region called the transfer region (Komano et al., 1990; Zatyka and Thomas, 1998; Koraimann and Wagner, 2014; Singh and Meijer, 2014). To minimize the host burden, expression of these genes is tightly regulated, and conjugation is only switched on under conditions that favor transfer of the conjugative element (Komano et al., 1990; Zatyka and Thomas, 1998; Koraimann and Wagner, 2014; Singh and Meijer, 2014). The majority of the genes are co-transcribed from a common promoter (Frost et al., 1994), which is activated by the two-component response regulator, ArcA, and the plasmid-specific positive regulator, TraJ (Silverman et al., 1991; Frost and Koraimann, 2010). IncI1 plasmids, such as R64, ColIb-P9 and R144 use a conjugation system very similar to that of the IncF plasmids, but with distinctive differences (Komano et al., 1990, 2000). They encode for two types of sex pili, a thin flexible pilus and a thick rigid pilus, both of which are antigenically and morphologically different from the IncF pilus (Komano et al., 1990, 2000), and the *tra-*region consists of approximately 30 transfer-related genes, the products of which share the same principal function as their IncF-encoded counterparts (Komano et al., 2000; Frost and Koraimann, 2010). IncI1 plasmids carry the *sog* gene which encodes DNA primase which is responsible for suppressing *dnaG* mutations in *Escherichia coli*, and IncI1 plasmids show a complex DNA rearrangement mediated by a unique structure designated shufflon (Komano et al., 1990).

Cephalosporinases, encoded by Extended Spectrum β-Lactamases (ESBLs), are a common cause of β-lactam resistance, particularly in *E. coli* and *Klebsiella pneumonia* (Lawley et al., 2003; Ilangovan et al., 2015). CTX-M-1, encoded from *bla*_CTX-*M-*1_, is among the most common ESBL gene, and this gene is often plasmid-encoded (Rodríguez et al., 2009). In the current study we used this gene, located on a naturally occurring IncI1 plasmid pTF2, to study the effect of cephalosporin treatment on the conjugation machinery and on conjugation frequencies. Importantly, we designed an experimental setup for measurement of conjugation frequency, in which we could separate conjugation rate from the power of selection by the antibiotic. Our results revealed an increased cephalosporin dependent conjugation frequency, thus contributing with novel insight to the on-going debate on the role of antimicrobial treatments in the spread of plasmid-encoded resistance.

## Materials and methods

### Bacterial strains and plasmids

The native IncI1 plasmid pTF2 carrying *bla*_CTX-*M-*1_ was isolated from a commensal *E. coli* of cattle origin. It had the typical IncI1 plasmid scaffold and did not harbor genes for resistance to additional therapeutic antimicrobials (accession number KJ563250). *E. coli* K-12 MG1655/pTF2 (Kjeldsen et al., 2015a), was used for protein and gene expression studies and as donor strain in conjugation experiments. *E. coli* MG1655/pTF2ΔCTX (Kjeldsen et al., 2015b) containing a non-coding sequence (NCS) instead of *bla*_CTX-*M-*1_ on the IncI1 plasmid pTF2 was used as a control strain. The NCS was constructed by randomly shuffling the sequence of *bla*_CTX-*M-*1_(Kjeldsen et al., 2015b), thus creating a sequence of the exact same length as the original gene, but with no open reading frame. Additionally, a strain (*E. coli* MG1655/CTX-M-1/pTF2ΔCTX), containing a NCS instead of *bla*_CTX-*M-*1_ on the IncI1 plasmid pTF2, and with CTX-M-1 encoded on the chromosome, was constructed in the current study by conjugating the pTF2ΔCTX plasmid from *E. coli* MG1655/pTF2ΔCTX into *E. coli* MG1655/CTX-M-1 (Kjeldsen et al., 2015a), which carries the *bla*_CTX-*M-*1_ on the chromosome. Strains and plasmids used are listed in Table 1. Screening for plasmid carrying strains among CTX resistant strains was done by PCR directed against *traU*. Primer sequences are shown in Supplementary Table S1. A rifampicin-resistant strain of *E. coli* J53-2 was used as recipient in conjugation experiment.

**Table 1:** Strains and plasmids used in the study.

**Name**	**Genotype**	**References**
**PLASMID**		
pTF2^a^	*bla*_CTX-*M-*1_ containing IncI1 plasmid pTF2 (amp^r^)	Kjeldsen et al., 2015a
pTF2ΔCTX	pTF2 with non-coding sequence inserted instead of CTX-M-1	Kjeldsen et al., 2015b
**STRAIN**		
MG1655/pTF2	*E. coli* MG1655/pTF2 (amp^r^)	Kjeldsen et al., 2015a
MG1655/pTF2ΔCTX	*E. coli* MG1655/pTF2ΔCTX	Kjeldsen et al., 2015b
MG1655/CTX-M-1	*E. coli* MG1655Δ*YbeM*::CTX-M-1 (amp^r^)	Kjeldsen et al., 2015a
MG1655/CTX-M-1/pTF2ΔCTX	*E. coli* MG1655Δ*YbeM*::CTX-M-1/pFT2ΔCTX (amp^r^)	This work
J53-2	*E. coli* J53-2, recipient strain (rif^r^)	Appelbaum et al., 1972

a *Accession number KJ563250*.

### Growth conditions

For protein and gene expression analysis, strains were grown in 250 mL flasks containing 100 mL of Mueller Hinton II (MH-2) broth (Sigma, Copenhagen, Denmark) at 37°C with shaking at 225 rpm. In addition to growth in antibiotic free medium, the medium was supplemented with two different concentrations of cefotaxime (CTX) (Sigma, Copenhagen, Denmark) representing $\frac{1}{2}$ MIC of the *bla*_CTX-*M-*1_ strain (126 mg/L) and 1/100 MIC of the *E. coli* K-12 MG1655 strain (0.016 mg/L) (Blattner et al., 1997). The high CTX concentration is within the range of therapeutic concentrations, as previously reported (Fu et al., 1979; Raddatz et al., 1995). Pre-cultures grown for 2 h at 37°C and 225 rpm were used to inoculate the cultures to a final cell density of 10^5^ CFU/mL. Growth was monitored by measuring optical density (OD_600_), and samples were taken in the exponential growth phase (OD_600_ = 0.5).

Growth of cells for measurement of effects of short time CTX treatment was performed by growing cultures, inoculated as described above, until they reached a cell density at OD_600_ = 0.5. At this point they were treated with 0 mg/L or 126 mg/L CTX for 5, 10, and 20 min before samples were taken for RNA extraction. In parallel, CFU of cultures were determined by plating 10-fold dilutions on LB-agar plates. Counts were made after incubation for 18 h at 37°C. All experiments were performed in biological duplicates or triplicate with two-three technical replicates each.

### Proteomics

#### Reduction, alkylation and proteolytic digestion

The culture samples were harvested and resuspendend in 300 μL sonication buffer [6 M urea, 2 M thiourea, 100 mM ammonium bicarbonate (all from Sigma, Copenhagen, Denmark)] pH 8.0 supplemented with EDTA-free protease inhibitor cocktail (complete ULTRA tablets, Roche, Hvidovre, Denmark). The resuspended cell pellets were sonicated on ice seven times for 15 s each at 70 W. Proteins in cell lysate (typically 100 μL) was reduced in 10 mM dithiothreitol (DTT) for 1 h at 25°C and alkylated with 50 mM iodoacetamide for 40 min at 25°C in the dark. Each sample was diluted 1:10 with 50 mM triethylammonium bicarbonate (TEAB) pH 8.0 and proteins were digested with 2% (w/w) trypsin for 16 h at 25°C. The peptide suspension was desalted using Oasis HLB Plus short cartridges (Waters, Hedehusene, Denmark) as recommended by the manufacturer and finally dried by vacuum centrifugation and stored at −20°C.

#### iTRAQ labeling

iTRAQ labeling was performed with the 4-plex iTRAQ reagents as recommend by the manufacturer (Applied Biosystems, Naerum, Denmark). Briefly, 10 μg peptide digest (as determined by amino acid analysis) was labeled at room temperature for 1 h after which 4 μL from each mass tag reaction was withdrawn, combined and analyzed by MALDI-TOF/TOF (UltraFlex II, Bruker Daltonics, Bremen) in order to estimate the relative ratios of each label. Appropriate samples amounts were pooled and dried by vacuum centrifugation. The iTRAQ sample was re-suspended in 50 μL 0.1% trifluoroacetic acid (TFA), desalted using Poros R3 micro-columns (Rappsilber et al., 2007), dried by vacuum centrifugation and stored at −20°C.

#### Liquid chromatography tandem mass spectrometry (LC–MS/MS)

Peptides were analyzed by an Easy-nLC and nanospray source (Thermo Fisher Scientific, Bremen, Germany) coupled with a Q-Exactive Plus mass spectrometer (Thermo Fisher Scientific, Bremen, Germany). Approximately 1 μg of labeled peptide (5 μL) was reconstituted in 0.1% formic acid and loaded onto a trap column at 250 bar [2 cm length, 100 μm inner diameter, ReproSil, C18 AQ 5 μm 120 Å pore (Dr. Maisch, Ammerbuch, Germany)] vented to waste via a micro-tee and eluted across a fritless analytical in-house packed resolving column (17 cm length, 75 μm inner diameter, ReproSil, C18 AQ 3 μm 120 Å pore) with a 107 min gradient of 0–30% LC-MS buffer B (LC-MS buffer A: 0.1% formic acid; LC-MS buffer B: 0.1% formic acid, 95% ACN) using a flow rate of 300 nL/min. Instrument method consisted of one survey scan (AGC target value: 1e6; R = 70 K; maximum ion time: 120 ms; mass range: 400–1,400 m/z) followed by data-dependent tandem mass spectra on the top 12 most abundant precursor ions [isolation width: 1.6 m/z; HCD collision energy (NCE): 32; MS1 signal threshold: 2e4; AGC MS2 target value: 1e6; maximum MS/MS ion time: 200 ms; dynamic exclusion: repeat count of 1, maximum exclusion list size, 20 s wide in time, ±10 ppm wide in m/z; doubly-charged precursors only; minimum signal threshold of 10,000].

#### Proteomics data process and database search

The LC-MS/MS data were processed with Proteome Discoverer (Version 1.4.1.14, Thermo Fisher Scientific, Bremen, Germany) and subjected to database searching using an in-house Mascot server (Version 2.2.04, Matrix Science Ltd., London, UK). Database searches were performed with the following parameters: Database, NCBI sub-taxonomy *E. coli*; Trypsin as the enzyme allowing a maximum of one missed cleavages sites; Carbomidomethylation of Cys as fixed modification; Deamidation of Asn and Gln; Oxidation of Met allowed as variable modification; iTRAQ was set as variable modification. Precursor and fragment mass tolerance were set to 10 ppm and 0.05 Da, respectively. Precursor mass range set from 350 to 7,000 Da. Protein identification was based on at least two unique peptides. False discovery rate was set to 1% at protein level using the Percolator algorithm. iTRAQ quantification was performed using Proteome Discoverer. The ratios were normalized against the protein median. The proteomics were performed in biological duplicates using two technical replicates. A threshold of 1.5 was used for identification of up-regulated proteins. Please use project number PXD006679 to access all generated MS/MS data stored in the PRIDE archive (https://www.ebi.ac.uk/pride/archive/).

### RNA extraction

At each sampling point from the proteomic study, a volume of 0.5 mL cell sample was mixed with 1 mL of RNAlater (Ambion®, Naerum, Denmark) according to the manufacturer's instructions and stored for immediate stabilization and protection of the RNA. A FastPrep cell disrupter system (Qbiogene, Illkirch, France) and an RNeasy Mini kit (Qiagen, Sollentuna, Sweden) was used to extract total RNA by mechanical disruption. Quantity of the extracted RNA was determined by A_260_ measurements and purity by A_260_/A_280_ ratio measurements using a NanoDrop 1000 spectrophotometer (Thermo Scientific, Hvidovre, Denmark). The total RNA was treated with TURBO™ DNase (2 U/μL) (Ambion®, Naerum, Denmark).

### Reverse transcribed-quantitative real time polymerase chain reaction (RT-qPCR)

One hundred and fifty nanograms of purified RNA were reversed transcribed with the High Capacity cDNA Reverse Transcription Kit (Life Technologies, Naerum, Denmark) according to the manufacturers' instructions. qPCR was performed essentially as described by Pfaffl, using a LightCycler 96 (Roche, Hvidovre, Denmark) (Pfaffl, 2001). The genes *gapA* and *nusG* were used as reference genes based on validation performed in another study (Kjeldsen et al., 2015a). The 2^−ΔΔCT^ method was used, however corrected for different primer efficiencies and multiple reference genes (Pfaffl, 2001). Two independent biological replicates were performed using two technical replicates. Primer sequences can be seen in Supplementary Table S1.

### Conjugation experiment

MG1655/pTF2 was used as donor and J53-2 as recipient strain in the conjugation experiments aiming to determine the conjugation transfer frequency when the CTX-M-1 gene was located on the IncI1 plasmid pTF2. MG1655/pTF2 was grown with and without CTX (126 mg/L) up to an OD_600_ = 0.5. In order to remove antibiotics from the donor strains, cells were washed in LB medium by centrifugation at 1,000 g for 3 min at room temperature, and then resuspended in fresh LB medium and adjusted to OD_600_ = 0.5. The recipient was grown without CTX to the same OD. The conjugations were performed on Difco™ lysogeny broth (LB) agar plates (Becton, Dickinson, Albertslund, Denmark) with filters (0.22 μM, Millipore, Copenhagen, Denmark) at 37°C. Donor (grown with and without antibiotics) and recipient were mixed in a 1:1 ratio to a final volume of 200 μl on the filters. This approach ensured that none of the antimicrobial that had been used in the pre-growth was present during the conjugation experiment. Mixtures were incubated at 37°C without shaking and conjugation was performed for 30 and 60 min. The bacterial material was washed of the filters using isotonic NaCl and the mixture was vortexed to stop the conjugation process. A dilution series was made and the mixture was plated onto LB agar plates containing 5 mg/L CTX (for quantifying donor+transconjugants), 50 mg/L rifampicin (for quantifying recipient+transconjugants) or 5 mg/L CTX and 50 mg/L rifampicin (for quantifying transconjugants only) and incubated overnight at 37°C. The conjugation frequency was calculated as transconjugants divided by number of donors times 100.

To confirm the counting of transconjugants by an independent method, colonies growing on LB agar plates containing 50 mg/L rifampicin (recipient and transconjugants) were scraped off and DNA was extracted using DNeasy Blood & Tissue Kit (Qiagen, Hilden, Germany). The concentrations and the quantity of the extracted DNA were determined by a NanoDrop 1000 spectrophotometer (Thermo Scientific, Hvidovre, Denmark). The number of transconjugants was determined by qPCR using previously published primers targeting 16s rDNA as the reference gene (Suzuki et al., 2000; Luprano et al., 2016) for number of bacteria, and primers targeting *traF* (Supplementary Table S1) to count the number of transconjugants. Note that this was done on DNA level, and thus the fact that *traF* is up-regulated in the presence of high concentrations of CTX is irrelevant.

*E. coli* MG1655/CTX-M-1/pTF2ΔCTX was used as donor and J53-2 was used as recipient to determine the conjugation transfer frequency when the CTX-M-1 gene was located on the chromosome. *E. coli* MG1655/CTX-M-1/pTF2ΔCTX was grown with and without CTX (84 mg/L = $\frac{1}{2}$ MIC of this strains) up to an OD_600_ = 0.5. In order to remove antibiotics from the donor strains, an additional washing step was also performed as mentioned above. The recipient was grown without CTX to the same OD_600_, and the conjugation experiments were performed for 30 and 60 min as described above. Antibiotics used in this study were purchased from Sigma (Copenhagen, Denmark). The experiments were performed in at least biological duplicates with two technical replicates each.

### Statistical analysis

Statistical analysis was performed using GraphPad prism version 6. Protein expression and gene expression was compared for the same protein/gene between conditions using One way ANOVA with Bonferreti's correction for multiple comparisons (between 0, 1/100 MIC and ½ MIC concentrations of antibiotics). Comparison of conjugation frequencies with and without added antimicrobial was performed by students *t*-test with Welch's correction. A *P* < 0.05 was considered significant.

## Results

**Figure 1:**
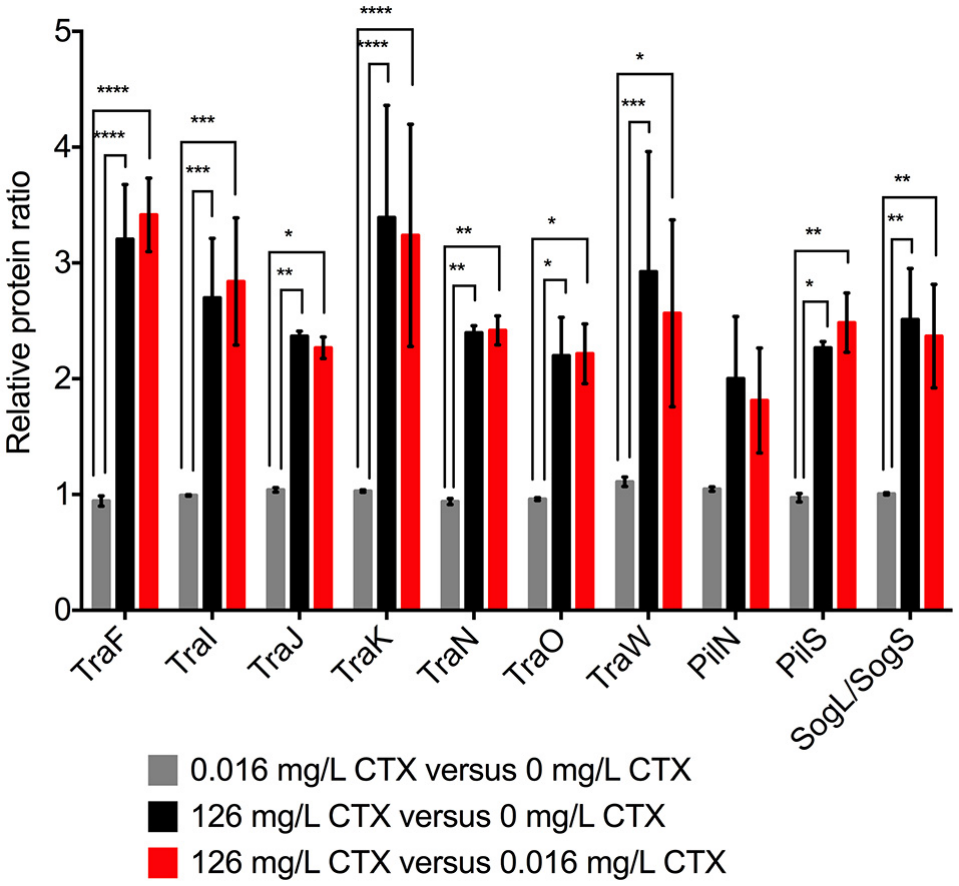
Relative protein ratios for proteins involved in conjugation for MG1655/pTF2. Relative protein levels were determined by iTRAQ. Cell samples grown at no (0 mg/L), sub-therapeutic (0.016 mg/L), and therapeutic (126 mg/L) concentrations of cefotaxime (CTX) were used for proteomics. The results shown are from two biological replicates with two technical replicates each. The data shown represents the mean and the error bars represent standard deviations. The stars indicate statistical significance at different levels: ^*^P ≤ 0.05, ^**^P ≤ 0.01, ^***^P ≤ 0.001, and ^****^P ≤ 0.0001.

A likely mechanism for antimicrobials to promote an increase in the number of conjugation events is to promote up-regulation of the T4S responsible for the conjugative transfer. In order to study whether the 3rd generation cephalosporin, CTX, affected expression of the conjugation apparatus of the IncI1 plasmid pTF2, we performed iTRAQ-based quantitative proteome analysis. The relative protein levels of *E. coli* strain MG1655/pTF2, carrying *bla*_CTX-*M-*1_ on pTF2, exposed to low (0.016 mg/L) and high (126 mg/L) concentrations of CTX, respectively, was determined relative to the isogenic control strain grown without CTX. The results showed that Tra-proteins and other conjugation related proteins were significantly up-regulated in the presence of high levels of CTX (One-way ANOVA, 0.0001 < *P* < 0.05 depending on the protein), while bacteria grown at the low concentrations of CTX showed insignificant differences in the proteome compared to the untreated control (One-Way ANOVA with correction for multiple comparison, *p* > 0.05). Nine out of ten plasmid transfer-related proteins detected in the analysis were up-regulated 2- to 3-fold, while the remaining protein (PilN) was up-regulated nearly 2-fold (Figure 1). Apart from the CTX-M-1 β-lactamase and a predicted HEAT domain containing protein (accession number: 585322259), no other proteins were found to be significantly up regulated as a result of CTX treatment. The full proteome data set is presented in Supplementary Dataset S1.

**Figure 2:**
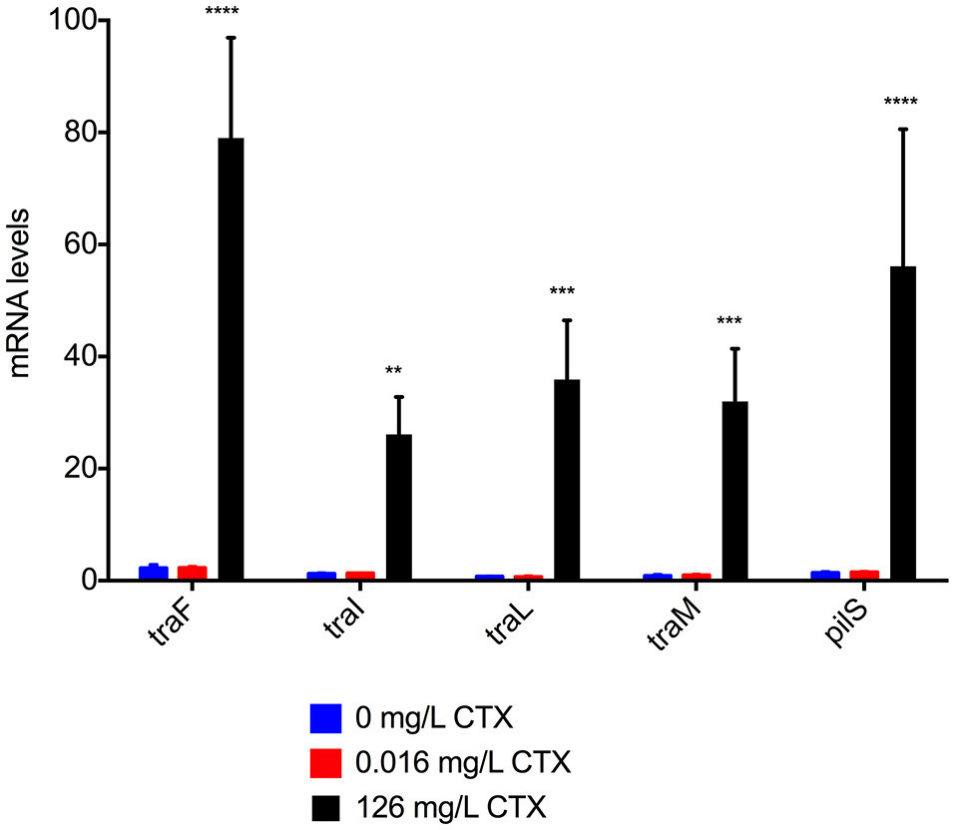
Expression of five selected transfer genes involved in conjugation in MG1655/pTF2. Fold change in mRNA levels were determined by qPCR. Cell samples grown at no (0 mg/L), sub-therapeutic (0.016 mg/L), and therapeutic (126 mg/L) concentrations of cefotaxime (CTX) were used for qPCR. Two independent replicates including two technical replicates each were performed; the data shown represents the mean and the error bars represent standard deviations. The data have been normalized to two validated reference genes, *gapA* and *nusG*. The stars indicate significant difference between 126 mg/L CTX and the other data points within each gene. The stars indicate statistical significance at different levels: ^**^P ≤ 0.01, ^***^P ≤ 0.001, and ^****^P ≤ 0.0001.

The up-regulation of transfer proteins in response to high doses of CTX was confirmed at the transcriptional level for five selected genes using RT-qPCR analysis (Figure 2). These genes were selected to represent the pilus synthesis and assembly system (*traF* and *traL*), the DNA transfer system (*traI* and *traM*) and the structural components of the pilus (*pilS*). Significant up-regulation (30- to 80-fold, One-Way ANOVA, 0.0001 < *P* < 0.001 depending on the gene) was observed when the bacterium was treated with high CTX concentrations during growth. In contrast, low CTX concentrations had no significant effect on conjugation gene transcription (One-Way ANOVA, *p* > 0.05). Thus treatment with high concentration of CTX caused up-regulation of the conjugation transfer system both at the transcriptional and translational level.

**Figure 3:**
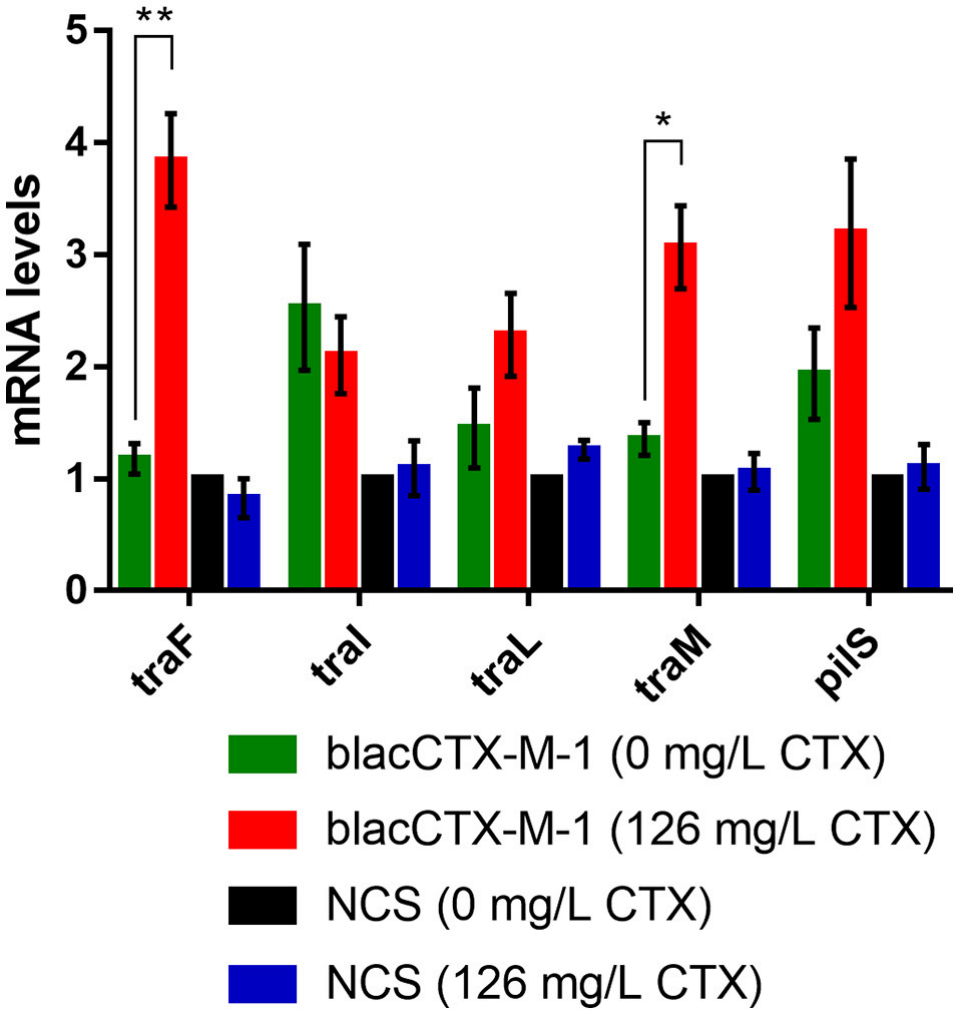
Expression of selected genes involved in conjugation in two strains, MG1655/pTF2 (*bla*_CTX-*M-*1_) and MG1655/pTF2ΔCTX (NCS) after treatment with CTX for short time. Relative mRNA levels were determined by qPCR. Cell samples were grown to exponential phase and then exposed to no (0 mg/L) or therapeutic concentrations of CTX (126 mg/L) for 20 min. The expression data was normalized to two validated reference genes, *gapA* and *nusG*. The results shown are means from two biological replicates with two technical replicates each and the error bars represent standard deviations. The stars indicate statistical significance at different levels: ^*^P < 0.05, ^**^P ≤ 0.01.

To investigate if the induction of transfer gene expression depended on the presence of the antibiotic resistance gene, *bla*_CTX-*M-*1_, a strain carrying a NCS in the plasmid instead of the resistance gene, MG1655/pTF2ΔCTX, was treated with a high concentration of CTX (126 mg/L) for a short period of time (20 min). Time kill curve established that at this time point, CFU of MG1655/pTF2ΔCTX grown in the presence of CTX did not differ significantly from CFU of the same strain grown without CTX, while at later time point it was significantly reduced (Supplementary Figure S1). The results showed that *tra-*gene mRNA levels in this strain were not different from the non-CTX treated control (*t*-test, *p* > 0.05), whereas the expression of the transfer genes *traF* and *traM* was significantly induced in the *bla*_CTX-*M-*1_ encoding isogenic strain after this 20 min of CTX treatment (Figure 3; *t*-test, *p* = 0.0051 and *p* = 0.0132). Expression of *pilS* and *traL* was not significantly up-regulated at this time point, but an increasing trend was seen. *traI* did not show up-regulation at this time point. A similar difference, yet not so pronounced, was observed at 5 and 10 min samples (data not shown). Together the results showed that up-regulation of *tra-*genes due to treatment with CTX was dependent of the presence of the resistance gene, *bla*_CTX-*M-*1_ in the strain. In order to rule out that the up-regulation was caused by a change in copy number of the plasmid relative to the chromosome, we carried out a qPCR based analysis. The ratio between a plasmid gene (*traF*) and *dxs* in the chromosome did not change significantly due to treatment with CTX in neither MG1655/pTF2 nor MG1655/CTXM-1/pTF2ΔCTX (Supplementary Figure S2).

**Figure 4:**
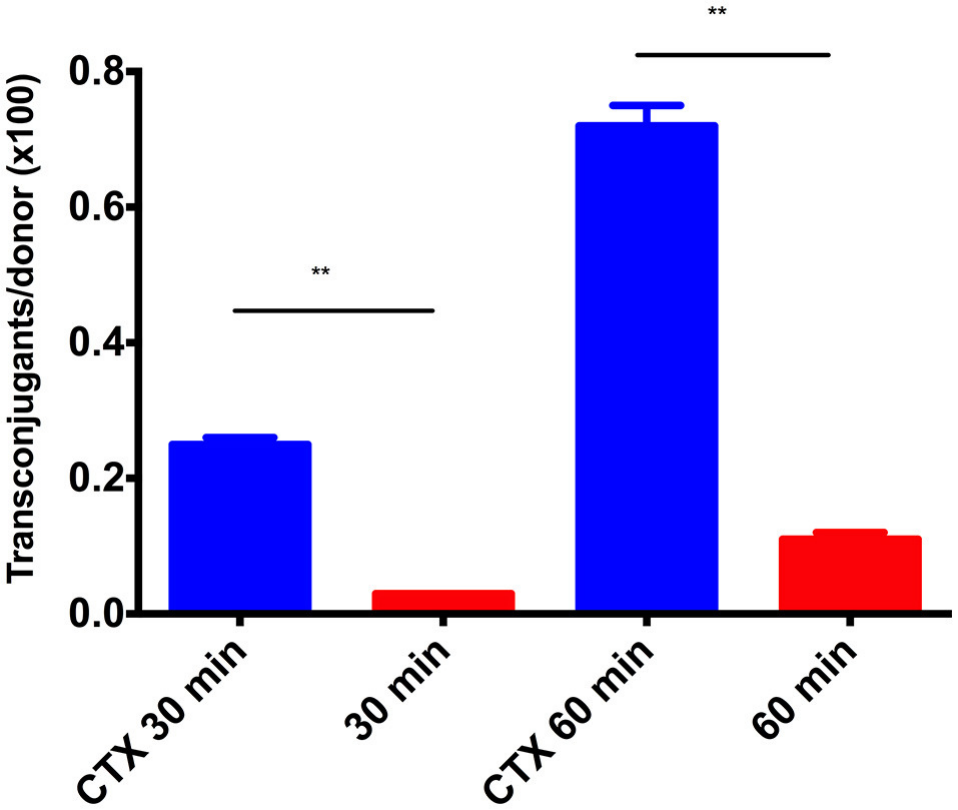
Conjugation-transfer-frequency for the plasmid pTF2 in MG1655/pTF2 grown with (bar CTX) or without CTX (bar no CTX) before the conjugation experiment. The results shown are from two biological replicates and the error bars represent standard deviations. The stars indicate statistical significance at level: ^**^P ≤ 0.01.

**Figure 5:**
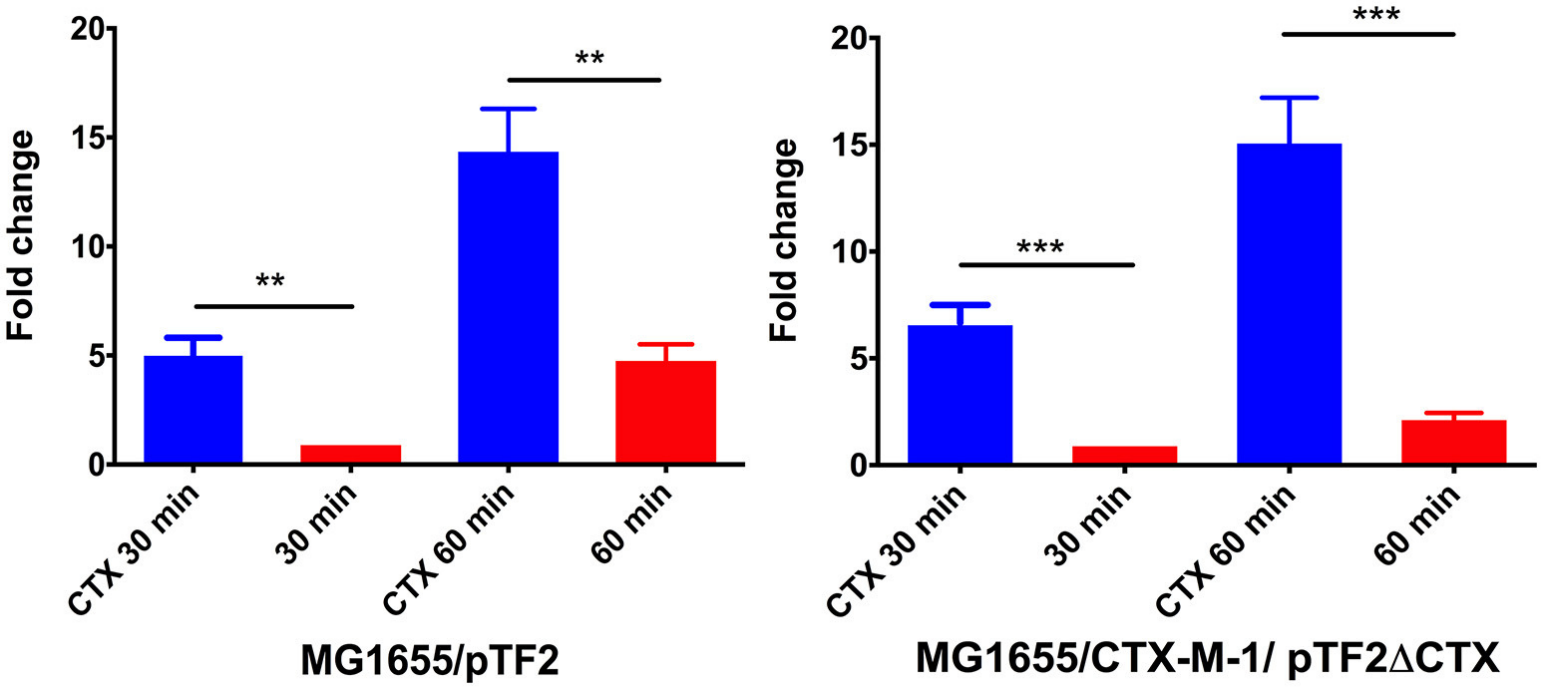
Conjugation-transfer-frequencies for MG1655/pTF2 **(Left)** and MG1655/CTX-M-1/pTF2ΔCTX with the CTX-M-1 gene located on the chromosome **(Right)** as measured by qPCR. The donor strains, MG1655/pTF2 or MG1655/CTX-M-1/pTF2ΔCTX, were grown with or without CTX before the conjugation experiment, and 30 and 60 min refers to the time the donor and recipient were having cell contact before the number of trans-conjugants was determined. The results shown are from three biological replicates and the error bars represent standard deviations. The stars indicate statistical significance at level: ^**^P ≤ 0.01, ^***^P ≤ 0.001.

In order to investigate whether up-regulation of the plasmid transfer system would lead to increased number of conjugation events, a conjugation experiment was performed in which we separated antibiotic treatment from the conjugation and the following selection for transconjugants. The MG1655/pTF2 strain was grown in the presence of CTX under the same conditions as for the proteome analysis, allowing the expression of conjugative transfer proteins. Conjugation was then performed by filter-mating for 30 or 60 min on agar plates without antimicrobials, after which conjugation was interrupted by centrifugation, before transconjugants were selected. A significantly increased number of transconjugants, corresponding to 8.4- and 6.6-fold higher conjugation transfer frequencies, were seen after 30 and 60 min of cell contact compared to conjugation experiments in which the donor was not grown in the presence of CTX before the conjugation (*t*-test, *p* < 0.01; Figure 4). The increased conjugation transfer frequencies were confirmed by performing the same experiment but this time quantifying the number of plasmids in the different pools relative to the number of bacteria by qPCR (5.0- and 3.1-fold higher transfer frequencies, *t*-test, *p* = 0.0037 and *p* = 0.0046; Figure 5).

The expression studies revealed that the up-regulation of *tra-*genes was dependent on the presence of *bla*_CTX-*M-*1_ in the strain. In order to determine whether *bla*_CTX-*M-*1_ had to be located on the resistance plasmid to affect conjugation efficacy, another conjugation experiment was performed using the strain MG1655/CTX-M-1/pTF2ΔCTX. This strain carried the CTX-M-1 encoding gene on the chromosome and a non-coding region instead of the *bla*_CTX-*M-*1_ on the IncI1 plasmid pTF2. The experiment showed that the CTX treatment also caused increased conjugation transfer frequency in this strain with 6.7- and 6.9-fold higher conjugation transfer frequencies after 30 and 60 min of cell contact, compared to conjugation without pre-growth in the presence of CTX (*t*-test, *P* = 0.0003 and *P* = 0.0004; Figure 5).

No proteins associated with the SOS response, such as LexA or RecA were significantly upregulate in the proteome of MG1655/pTF2 grown in the presence of high concentrations of CTX when compared to growth without CTX. This was further supported by RT-qPCR analysis showing that expression of the typical SOS-responsive genes, *recA* and *sfiA*, were not affected significantly by growth in the presence of CTX (Supplementary Figure S3).

## Discussion

It is well-established that conjugation plays a major role in the global spread of antibiotic resistance, and evidence implicating antibiotic treatment as a stimulating factor for conjugation transfer has been presented (Aminov, 2011; Andersson and Hughes, 2014). It remains unclear if antimicrobial treatment affects the conjugation efficiency of resistance plasmids directly, or merely selects for bacteria that have become resistant due to conjugation, and a recent study by Lopatkin et al. (2016a) points to a lack of conclusive evidence on this issue, since conjugation experiments are generally carried out in the presence of antimicrobials. Thus the experimental setup does not allow separation of conjugation frequency from selection of resistant bacteria. This may suggest that the contribution of antibiotics to the promotion of HGT may have been overestimated (Lopatkin et al., 2016a,b). In the present study, we avoided the post-conjugational selection effect by pre-growing donor cells in CTX, and then performing the conjugation in an antimicrobial free environment. Furthermore, the conjugation process was only allowed for 30 or 60 min, before vortexing and centrifugation stopped the process.

We found that genes and corresponding proteins involved in the conjugation apparatus of an IncI1 resistance plasmid pTF2 encoding CTX-M-1 were up-regulated in the presence of high concentrations of CTX. It was furthermore demonstrated that this up-regulation was associated with an increase in conjugation frequency in the absence of antimicrobials during the actual conjugation events. This evidence strongly suggests that antimicrobials, at least of this type, affects conjugation efficiency directly in addition to selecting for transconjugants. Differences to previous observations (Lopatkin et al., 2016a,b) may be due to other combinations of plasmid and drugs studied, but it may also rely on our changed experimental setup, and the fact that high concentrations of drugs, corresponding to therapeutic concentrations, were used here, while previous studies have focused on lower concentrations of antimicrobials. Induction of conjugation transfer gene expression has previously been described after treatment of *A. hydrofila* with sub-inhibitory concentrations of tetracycline, trimethoprim and flumequine (Cantas et al., 2012). Similarly, transfer genes encoded by the *Bacteroides* conjugative transposon CTnDOT were found to be induced by tetracycline and down-regulated in the absence of the antibiotic (Whittle et al., 2002). Here we report such a response for the first time for an ESBL plasmid in *E. coli* treated with therapeutically relevant concentrations of an antimicrobial substance.

The experiments were also carried out with a strain in which the *bla*_CTX-*M-*1_ gene was replaced with a random non-coding sequence of the same size, maintaining the promoter region. No induction of *tra-*genes in response to CTX treatment was seen in this strain, demonstrating that regulation was dependent on the presence of the *bla*_CTX-*M-*1_ gene itself, or production of the gene product, CTX-M-1. Conjugation efficacy was still significantly increased when the *bla*_CTX-*M-*1_ gene was located on the chromosome, showing that the resistance gene can contribute to increased conjugation frequency when located *in trans*. The production of CTX-M-1 was highly induced by CTX treatment, confirming previous reports that expression of CTX-M-1 is dependent on treatment with the drug (Kjeldsen et al., 2015a).

The exact mechanism by which the resistance gene influences conjugation frequency remains to be determined. Increased plasmid conjugative transfer has been reported as a consequence of the SOS response in *E. coli*, especially in relation to growth in the presence of low concentrations of antimicrobials (Sutton et al., 2000; Baharoglu et al., 2010; Cantas et al., 2012; Lopatkin et al., 2016b). The SOS-response is a widespread regulatory network induced by DNA damage, and induction of the SOS response has been suggested to promote the spread of mobile genetic elements (Úbeda et al., 2005; Maiques et al., 2006). None of the SOS response proteins were found to be up-regulated in the proteomic analysis of MG1655/pTF2 and the typical SOS-responsive genes, *recA* and *sfiA*, were not induced by growth in the presence of CTX. The lack of SOS response induction suggests that changes in conjugation observed in the current study differs from previously reported influences of antibiotic treatment on plasmid transfer efficiencies (Barr et al., 1986; Beaber et al., 2004; Baharoglu et al., 2010; Cantas et al., 2012). Conjugal transfer of plasmids from donor to recipient cells is a complex process. Others factor are involved in conjugal transfer apart from the SOS response. Miyazaki et al. (2012) reported that in absence of the stationary phase sigma factor RpoS in *Pseudomonas knackmussii*, integrative and conjugative element ICEclc transfer rates and activation of two key ICEclc promotors (P_int_ and P_inR_) decreased significantly in cells during stationary phase, suggesting that high RpoS levels are a prerequisite for activating the ICEclc promoter and thus ICEclc transfer in *P. knackmussii*.

In the present study, effect of antimicrobial treatment on conjugation frequency has been studied in a system, where selection of transconjugants by the antimicrobial can be ruled out, and where the effect of a therapeutically relevant concentration of an antimicrobial has been used. Further experiments are needed to uncover the underlying mechanism behind CTX-mediated conjugation induction and to determine if the observed effect applies to other plasmid types and other types of antibiotics. This is likely to include detailed studies of the regulation of the IncI1 transfer genes, as well as the transfer system of other plasmid types. Overall, the observations in the study clearly indicate that antibiotic treatment may not only select for antimicrobial resistant bacteria, but also promote increased conjugation frequency of resistance plasmids in the gut. It is important to determine which combination of cephalosporin treatment and plasmids/ESBL genes that show this response, since increased conjugation frequency will be an unwanted, adverse effect of treatment with these potent drugs.

## Author contributions

TM, LT, JM-J, and JO designed the experiments. TM, GL, AB, FL, LT, and SM carried out the experiments. TM, GL, and JO drafted the manuscript. All authors commented on and approved the final manuscript.

### Conflict of interest statement

The authors declare that the research was conducted in the absence of any commercial or financial relationships that could be construed as a potential conflict of interest.
