# Supplementary material for: Treatment with Cefotaxime Affects Expression of Conjugation Associated Proteins and Conjugation Transfer Frequency of an IncI1 Plasmid in Escherichia coli
Source: Front Microbiol. 2017 Nov 29;8:2365. doi: 10.3389/fmicb.2017.02365 (PMC5712592; doi:10.3389/fmicb.2017.02365)
Supplement: Supplementary file 1 [file Table1.pdf]

Supplementary Table S1. Primer sequences used for PCR.

**Gene**	**Primer sequences**
nusG	for: 5’-GTCCGTTCGCAGACTTTAAC-3’
	rev: 5’-GCTTTCTCAACCTGACTGAAG-3’
gapA	for: 5’-ACTGACTGGTATGGCGTTCC-3’
	rev: 5’-GTTGCAGCTTTTTCCAGACG-3’
pilS	for: 5’-AGGGGCTGCTAAAAGGTAGC-3’
	rev: 5’-CTGTCATGGTCTTGGGTACG-3’
traF	for: 5’-GACGTCGGAATTTCATTTGC -3’
	rev: 5’-TCCACACGCTGATATTTTGG -3’
traI	for: 5’-CGGGAAAGCACACTTAATGC-3’
	rev: 5’-CTGGCGTGATATGAGCTACG-3’
traL	for: 5’-ATGGTCACAACGTGAAAACG-3’
	rev: 5’-GAACTGGGGGAGGTTTATGC-3’
traM	for: 5’-GGAGTCAGAATGATGCAATGG-3’
	rev: 5’-AGGGAGGAGATCTGTGAACG-3’
recA	for: 5´-ACACCGGCGAGCAGGCACTGGAAA-3’
	rev:5´-ACGTGCCGCAAGGCCCATGTGA-3’
sfiA	for: 5’- CGGGAATGGGTTCAGGCATCTGGGC-3’
	rev: 5’- TGCCCGTGCGTAAAGCGCGAACCA-3’
traU	for: 5´- ATCTTCGGGTTTGTAGCGCA 3´,
	rev: 5´ - ACAGAAGACGGTTCCGCAAT 3´
16s rRNA	for: 5’-CGGTGAATACGTTCCCGG-3’
	rev: 5’- GGTTACCTTGTTACGACT-3’
dxs	for: 5’-CGAGAAACTGGCGATCCTTA-3’
	rev: 5’-CTTCATCAAGCGGTTTCACA-3’
