# Supplementary material for: Treatment with Cefotaxime Affects Expression of Conjugation Associated Proteins and Conjugation Transfer Frequency of an IncI1 Plasmid in Escherichia coli
Source: Front Microbiol. 2017 Nov 29;8:2365. doi: 10.3389/fmicb.2017.02365 (PMC5712592; doi:10.3389/fmicb.2017.02365)
Supplement: Supplementary file 3 [file Image1.pdf]

Supplementary Figures.

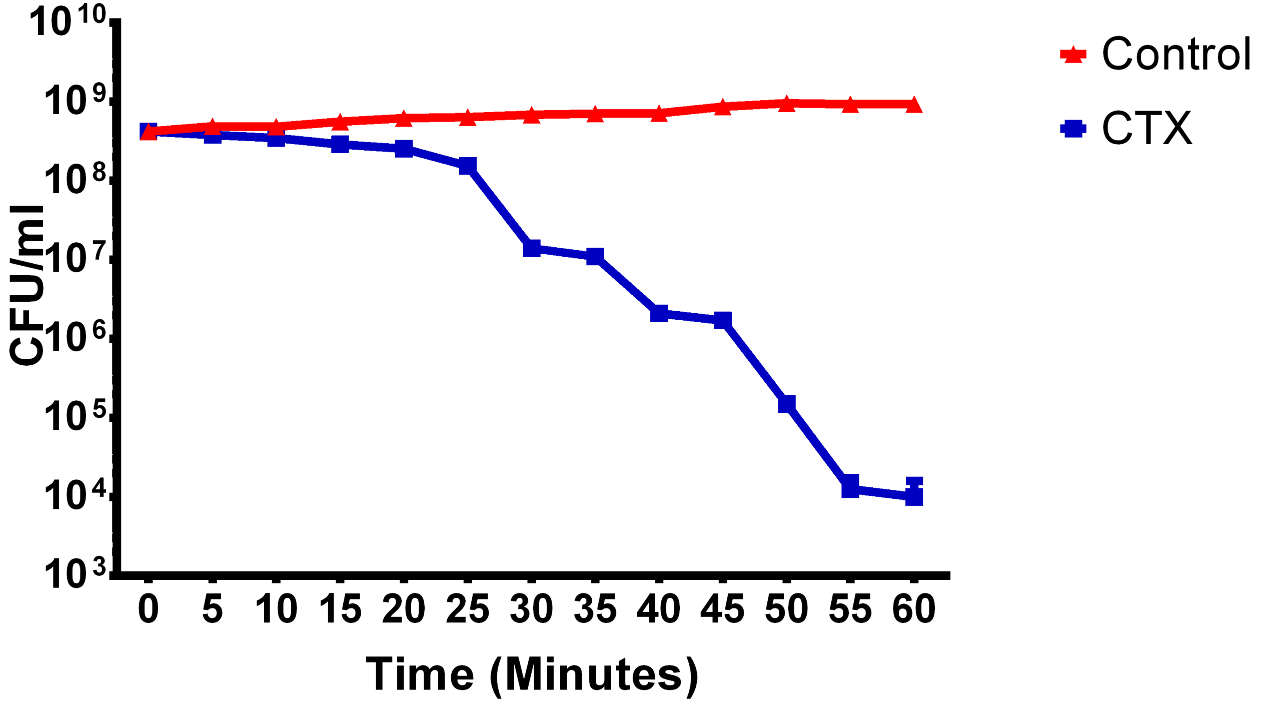

Supplementary figure S1. Time-kill curve of MG1655/ pTF2ΔCTX in LB in the presence of 126 mg/L CTX or without CTX (control).
CFU/ml was not statistically different at 20 min post inoculation.

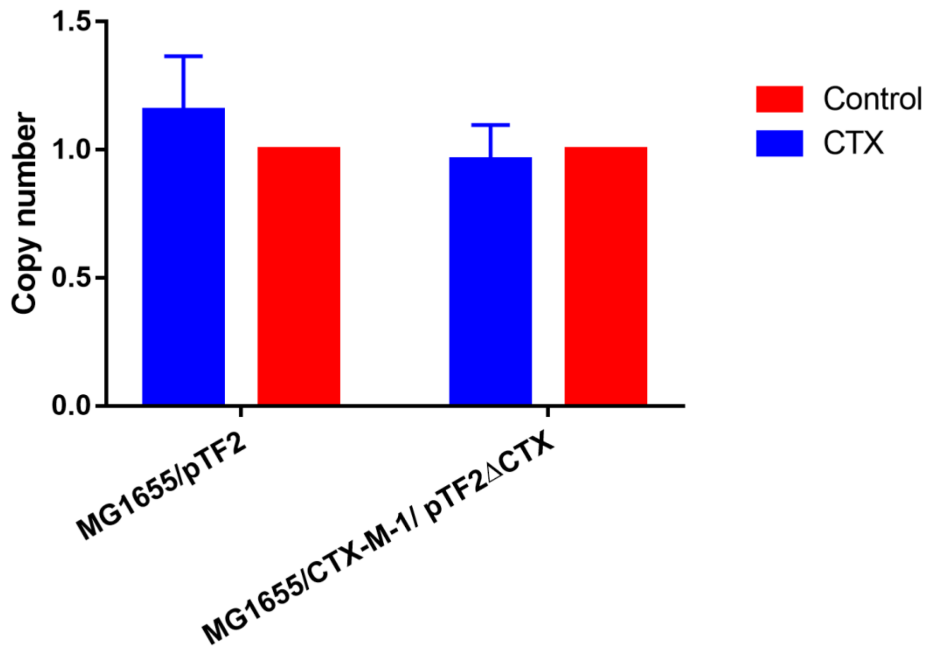

Supplementary Figure S2. Copy number analysis of pTF2 with and without *bla*$_{CTX\text{-}M\text{-}1}$ grown without (control) and with 126 mg/L CTX.

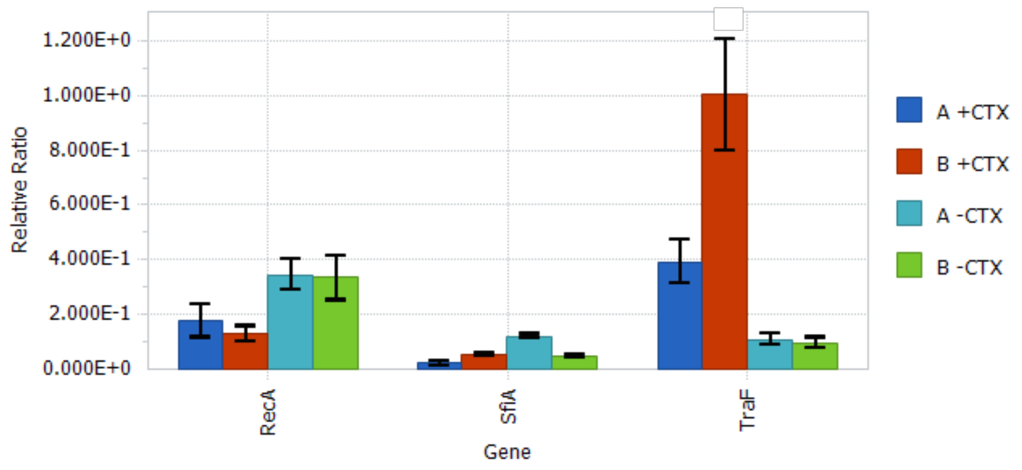

Supplementary Figure S3. Gene expression analysis of two SOS response genes (*recA* and *sfiA*) and *traF* in pTF2 grown with 126 mg/L CTX or without CTX. A and B denotes two biologically independent experiments.
